# Concentration-Dependent Modes of Melittin-Induced Membrane Destabilization: An Integration of Experimental and Computational Tools

**DOI:** 10.1021/acs.langmuir.6c02887

**Published:** 2026-09-09

**Authors:** Andrea Chacón Calderón, Pavel Zelenovskii, Márcio Soares, Ellen C. Wrobel, Osvaldo N. Oliveira Jr, Daniel Pereira, Mariana Sardo, Luís Mafra, Ana Barros-Timmons, Ildefonso Marín-Montesinos

**Affiliations:** † Department of Chemistry & CICECO−Aveiro Institute of Materials, 56062 University of Aveiro, Aveiro 3810-193, Portugal; ‡ Department of Physics & CICECO−Aveiro Institute of Materials, University of Aveiro, Aveiro 3810-193, Portugal; § São Carlos Institute of Physics, University of São Paulo, CP 369, São Carlos, São Paulo 13560-970, Brazil

## Abstract

Melittin is a well-known antimicrobial peptide whose mechanism of action is still not entirely understood. In this study, we combine complementary experimental techniques with molecular dynamics simulations to determine melittin-membrane interactions at different scales. Using 1,2-dioleoyl-*sn*-glycero-3-phosphoethanolamine (DOPE): 1,2-dipalmitoyl-*sn*-glycero-3-phospho-(1’-rac-glycerol) (DPPG) membranes that mimic the *Escherichia coli* inner membrane, this integrative approach reveals a clear transition in melittin’s mode of action with increasing peptide loading. At low concentrations, melittin primarily perturbs the membrane interface, inducing modest surface expansion and subtle lipid reorganization. At higher concentrations, the membrane response shifts toward increased hydration, lipid disorder, membrane thinning, and morphological remodeling. Simulations support these observations, linking concentration-dependent membrane responses to enhanced lipid mobility and stronger effects on anionic DPPG. Melittin, therefore, affects the membrane in a concentration-dependent manner, transitioning from surface-bound carpet-like permeabilization at low loading to transient pore formation and membrane reorganization at higher concentrations.

## Graphical abstract

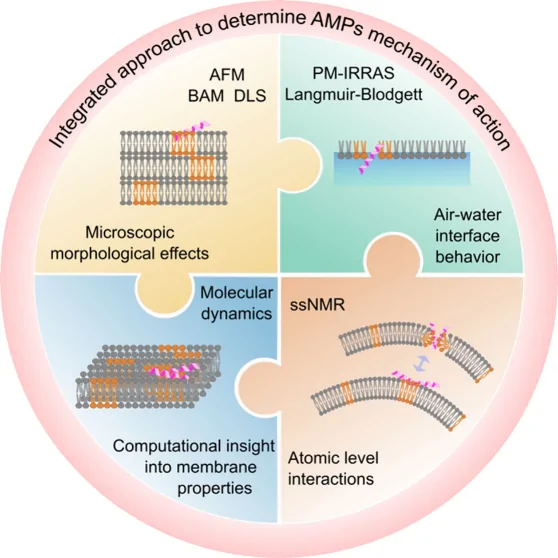

## 1 Introduction

Antimicrobial peptides (AMPs) are short molecules found in a wide range of bacteria, animals, and plants, serving as part of the host’s immune defense with a broad spectrum of activity against pathogenic bacteria, viruses, and fungi. AMPs possess an overall cationic charge and a significant proportion of hydrophobic residues,^1^ enabling selective interaction with the negatively charged microbial membranes and disrupting their integrity. Their primary mode of action involves membrane disruption, while secondarily targeting intracellular moieties and key cellular processes. Several mechanisms of action have been proposed,^2^ including pore formation (through the barrel-stave model and the toroidal-pore model), surface accumulation (carpet-like model), and membrane aggregation. AMPs are a promising alternative to conventional antibiotic therapy to address antibiotic resistance.^3^ Their rapid and multifaceted mechanisms of action make it difficult for pathogens to develop resistance, being advantageous in combating multidrug-resistant infections. Several AMPs have already reached clinical and commercial use, such as gramicidin,^4^ daptomycin,^5^ and polymyxins,^6^ while others, including pexiganan and omiganan,^5^ are in advanced stages of development for therapeutic applications.

Despite their promise, the natural diversity and versatility of these peptides mean their mechanisms of action can be difficult to define, limiting application, rational optimization, and translation. This is exemplified by melittin, a widely used reference AMP derived from bee venom^7^ whose membrane activity has been studied extensively, yet its mechanism is heavily condition-dependent. Melittin binds to the membrane surface via electrostatic interactions and, upon sufficient accumulation, adopts an amphipathic α-helical structure, inserting into the lipid bilayer and inducing structural perturbations.^8–10^ While melittin is commonly described as a pore-forming membrane-lytic peptide,^2^ key molecular uncertainties persist. It is still unclear which membrane properties (such as lipid composition and cholesterol content) shift the concentration thresholds for surface binding, insertion, and pore formation.^11^ Additionally, recent studies debate whether melittin-induced membrane permeabilization is dominated by equilibrium pore formation versus nonequilibrium/transient toroidal defects, and which conditions trigger one mode or the other.^12–14^ Mechanistic resolution is important because these regimes present distinct design opportunities and safety profiles. For example, parameters that promote cooperative membrane insertion and equilibrium pore formation enhance antibacterial and anticancer potency but may increase host-cell lysis due to reduced selectivity.^15^ On the other hand, shallow insertion, surface-binding to specific membrane components, or triggered pore formation increase the versatility of applications.^16,17^ Rational design of melittin-derived AMPs based on definitive mechanistic insight can make them optimal for clinical use.

Understanding the mechanisms by which AMPs interact with cellular membranes remains a significant challenge, largely due to the methodological constraints of early studies. The latter often relied on simplistic models, such as single-component lipid vesicles or basic antimicrobial assays, to infer the AMP activity. However, these methods often yielded conflicting conclusions, as is the case for melittin. For instance, while vesicle leakage assays initially suggested that melittin disrupts membranes solely by forming stable barrel-stave pores,^18^ later studies under different conditions proposed a detergent-like solubilization mechanism.^19,20^ Such discrepancies arise because simplistic models overlook critical variables (e.g., lipid composition, peptide concentration, and intracellular targeting), underscoring the need for more-comprehensive methodologies.

Herein, we investigate the interaction of melittin with a simplified model of the *E. coli* inner membrane, using the two majority components DOPE and DPPG. Given the goal of working with the most simple binary mixture, cardiolipin was excluded from the formulation. Two different lipid-to-peptide ratios (L:P 30:1 and 10:1) were studied across three model membranes, with complementary spectroscopic and microscopy techniques. DOPE and DPPG were used to produce: *i)* Langmuir monolayers to carry out surface pressure (π) versus area isotherms and BAM to assess morphological changes, with parallel PM-IRRAS measurements identifying key functional groups involved in air/water interface interactions; *ii)* thin lipid films analyzed by AFM to characterize melittin-induced nanoscale reorganization of lipid stacking in the dry state; and *iii)* lipid bilayers studied by DLS to evaluate the effect of melittin on size and aggregation of the lipid vesicles, and ssNMR to resolve atomic-level peptide-membrane interactions. This multitechnique strategy provides a comprehensive view of melittin membrane activity, correlating peptide structure with membrane composition and dynamics. Cross-validating results from interface-sensitive, nanoscale, and atomic-resolution methods, we overcome the limitations of single-technique approaches and establish a robust mechanistic framework for the membrane interactions with melittin.

## 2 Materials and Methods

### 2.1 Materials

Melittin from honey bee venom (>85%, M2272) was purchased from Sigma-Aldrich. The lipids used for preparation of the model membranes were 1,2-dipalmitoyl-*sn*-glycero-3-phospho-(1’-rac-glycerol) (DPPG) and 1,2-dioleoyl-*sn*-glycero-3-phosphoethanolamine (DOPE). They were supplied by Avanti Lipids Inc. and stored at −20 °C until utilization. Stock solutions with a final concentration of 10 mg mL^–1^ were prepared by dissolving DOPE in chloroform and DPPG in a 4:1 (v:v) chloroform:methanol mixture. The stock solutions were stored at −20 °C between uses. The buffer solution consisted of 5 mM *N*-2-hydroxyethylpiperazine-*N*-2-ethanesulfonic acid (HEPES) and 20 mM sodium chloride (NaCl) in water, adjusted to pH ∼ 7.2. The buffer was stored in sealed glass bottle in the fridge at 4 °C between uses.

### 2.2 Preparation of Liposomes

The liposomes used for the DLS and ssNMR studies were prepared using the thin film hydration method.^21^ The lipids were dissolved in chloroform, mixed in the desired ratios, and dried under a continuous stream of nitrogen to form a dry lipid film on the vial walls. Complete solvent evaporation was ensured by overnight vacuum drying. The lipid film was then hydrated with water or buffer solution, at a temperature above the highest phase transition temperature (*T*_m_) of the lipids in the sample, followed by ∼ 5 min of sonication to reduce aggregation. The lipid suspension was stirred until liposome formation, cooled, and stored at 4 °C overnight for complete hydration. For the samples containing melittin, the peptide was first solubilized in a chloroform/methanol mixture and added to the lipid solution before solvent evaporation. The solvent drying and lipid hydration steps were performed following the protocol described for the peptide-free samples.

### 2.3 Characterizations

#### 2.3.1 Dynamic Light Scattering (DLS)

The hydrodynamic diameter, particle size distribution, and polydispersity index of the vesicles were measured by DLS using a Zetasizer Nano-ZS equipment (Malvern Instruments). The measurements were performed at 25 °C with a backscattering angle of 175°.

#### 2.3.2 Atomic Force Microscopy (AFM)

Thin lipid films, with and without melittin, for AFM experiments were prepared on glass microscopy slides using a Spin Coater Model P6708D Series (Specialty Coating Systems). Spin-coating was performed by dropping 400 μL of the lipid solution on the slowly rotating glass slide followed by acceleration to 2000 rpm for 60 s. The samples were then stored in a desiccator under vacuum overnight to ensure complete solvent evaporation. The AFM characterization was performed with a Park HX-Hivac microscope (Park Systems) at low vacuum (air pressure below 1 mbar) to avoid the air humidity and room temperature. A silicon tip Multi75E-G (Budget Sensors) with a nominal resonance frequency 75 kHz and a force constant of 3 N m^–1^ was used. The scanning was performed in a noncontact mode at a resonance frequency of 81.7 kHz and an amplitude of free vibrations 15 nm. The set point was 80% of the free vibrations.

To measure the film thickness, the films were carefully scratched by a blade to avoid the glass substrate damage. Since spin-coating technique creates films of highly homogeneous thickness, one scratch in the middle of the sample has been done. AFM height imaging was performed to determine the film thickness. In total, 100–200 linear profiles have been measured across the scratch for each sample, and the average height values of the top and bottom parts of the scratches were calculated for each profile. The film thickness was determined for each profile as a difference between the average height values of the top and bottom parts of the profile, and, finally, the average thickness has been calculated. The results are presented in the form of mean ± STD.

#### 2.3.3 Langmuir Films, PM-IRRAS, and BAM

Langmuir films were prepared in a clean room using a Mini KSV Langmuir trough (KSV Instruments Ltd.). Surface pressure–area (π–A) isotherms were recorded at 295 K using a Wilhelmy plate (Whatman Chr1) connected to an electrobalance. Lipids and melittin stock solutions (1 mg mL^–1^) were prepared in chloroform/methanol. The films were formed by spreading the desired solution at the air–water interface using a microsyringe (Hamilton, 100 μL). The compressibility modulus (C_S_^–1^) was calculated from the compression isotherms using the following equation:

1 $${C_{S}}^{-1}=-A\left(\frac{d\pi }{dA}\right)$$

The collapse pressure (π_col_), lift-off area (A_LO_), and mean molecular area at 30 mN m^–1^ (A_30_) were determined directly from the compression isotherms.

The Langmuir monolayers were equilibrated for 10 min before being compressed to the desired surface pressure at a rate of 10 mm min^–1^. Polarization-modulation infrared reflection–absorption spectroscopy (PM-IRRAS) spectra were recorded with a KSV PMI 550 instrument (KSV Instruments Ltd.) over the Langmuir trough, with polarization between p and s modulated at high frequency. The incidence angle was set to 81°, and spectra were acquired at 30 mN m^–1^ with 600 scans and 8 cm^–1^ resolution, using the corresponding subphase spectrum as background. Brewster angle microscopy (BAM) images were recorded with a EP4 imaging ellipsometer (Accurion) at the air–water interface of a Langmuir Mini trough from KSV. The BAM instrument was fitted with an Ultraobjective camera, set at an incidence angle of 53.1°, with the polarizer at 10°, analyzer at 2°, and compensator at 0°. The images were corrected with Accurion’s Data Studio software.

#### 2.3.4 Solid-State Nuclear Magnetic Resonance (ssNMR)

The liposomes prepared by the thin-film method previously described were separated from the bulk aqueous phase by centrifugation and packed into a 4.0 mm ssNMR rotor, as described previously for this type of samples.^22^ ^1^H, ^13^C, and ^31^P spectra were acquired on a Bruker Avance III 400 spectrometer operating at B_0_ field of 9.4 T, with Larmor frequency of 400.130 MHz for ^1^H at a temperature of 300 K. All experiments were performed on double-resonance 4 mm Bruker magic-angle-spinning (MAS) probes at a MAS frequency of 10 kHz and at 300 K. Samples were packed into 4 mm MAS rotors with inserts and caps made of Kel-F. For the ^1^H single-pulse experiments, the initial 90-degree pulse had a duration of 3.0 μs and a recycle delay of 1.0 s. 1D ^13^C spectra were acquired under high-power proton decoupling using the SPINAL64 sequence, with a recycle delay of 4.0 s and a 90-degree pulse length of 4.0, 3.75, and 5.0 μs for the DOPE:DPPG, (DOPE:DPPG):MLT 30:1, and (DOPE:DPPG):MLT 10:1, respectively; the rf-field strength for the ^1^H decoupling pulses was 42 kHz. For ^13^C INEPT experiments, the ^1^H 90-degree pulse length was set to 5 μs; nonselective polarization transfer was achieved using an INEPT pulse sequence with evolution delays τ_1_ and τ_2_ of 2.0 and 1.2 ms, respectively. The recycle delay was 1.75 s, and ^1^H decoupling was employed at a rf-field strength of 45 kHz during signal acquisition.

For the 1D ^31^P experiments, also under high-power proton decoupling, the 90-degree pulse length was 4 μs, with recycle delays of 3.0 s, and with a rf-field strength for the ^1^H decoupling pulses of 76.5 kHz. The longitudinal relaxation time (T_1_) values for ^31^P were obtained using a saturation recovery pulse sequence. The ^31^P 90-degree pulse length was set to 4.0 μs employing a 1.0 s recycle delay, and a high-power proton decoupling pulse of 76.5 kHz of amplitude; 14 interpulse delays were used varying from 1 ms to 32 s. The different contributions for each ^31^P spectrum were deconvoluted using the Solids Lineshape Analysis module in TopSpin 4.2.0 program (Bruker), and the T_1_ fittings were done in the OriginPro9 2012 software package (OriginLab).

#### 2.3.5 Molecular Dynamics (MD)

The interaction of melittin with the model membrane was studied with molecular dynamics simulations for three different systems containing a fully hydrated lipid bilayer in the absence or presence of melittin peptides. In these systems, both upper and lower leaflets of the bilayer consisted of 100 DOPE molecules and 25 DPPG molecules, maintaining the 4:1 ratio used for the experimental analysis. For every system, a thick layer of water molecules was added to minimize the effect of interbilayer interactions, with 60 water molecules per lipid molecule. The systems consisted of I) membrane only, II) membrane with 2 melittin chains, and III) membrane with 4 melittins chains. The initial configuration was prepared using CHARMM-GUI.^23–27^ Each of the melittin peptides in α-helical conformation (PDB ID: 2MLT) was placed side-by-side on the bilayer-water interface of one of the leaflets. All simulations were performed using the GROMACS package (version 2023.2).^28^ The CHARMM36m force field was employed to model the melittin peptides and lipid molecules while the TIP3P model was used for water. The bilayers were equilibrated for 6 ns, and the production phase ran for 250 ns. For the production phase, the temperature was maintained at 323.15 K using a Nosé-Hoover thermostat. The pressure was set at 1 bar using a Parrinello–Rahman barostat, and a semi-isotropic coupling scheme was employed to control the pressure in the directions parallel and normal to the bilayer, independently. Electrostatic interactions were calculated with the particle mesh Ewald (PME) method. Lennard-Jones interactions were truncated at 1.2 nm with a smooth switching function applied in the range of 1.0 – 1.2 nm. The LINCS algorithm was used to constrain all bonds involving hydrogen atoms. The time step size was set to 2 fs. The density and order parameters for the acyl chains were calculated using the corresponding GROMACS scripts. The analyses of area per lipid, membrane thickness, and diffusion coefficient were performed with MDAnalysis,^29,30^ LiPyphilic,^31^ and freud.^32^ The snapshots were visualized using PyMOL.^33^

## 3 Results and Discussion

### 3.1 Impact of Melittin on Membrane Fluidity, Lipid Packing, and Structural Integrity

Langmuir monolayers composed of DOPE:DPPG in an 4:1 molar ratio were prepared to model a simplified *E. coli* inner membrane. Their interaction with melittin was studied at L:P molar ratios of 10:1 and 30:1. Compression isotherms and compressibility modulus of these monolayers were obtained by using the Langmuir–Blodgett technique. The shape of the π-A isotherms shown in Figure 1A indicate that no phase transition occurs for any of the monolayers, and all of them exist in the liquid-expanded phase,^34^ as evidenced by the maximum compressibility modulus (C_S_^–1^) below 100 mN m^–1^ (Figure 1B). The presence of melittin causes monolayer expansion, with lift-off areas increasing proportionally to the peptide concentration (Table 1). The consistent rise in mean molecular areas across all isotherms up to the collapse point suggests full integration of melittin into the monolayers.^35^ Despite this incorporation, the large-scale phase behavior of the monolayers at the air–water interface remains unchanged, with the monolayers staying in a liquid-expanded phase at both L:P ratios. The reduced collapse pressure at the 10:1 L:P ratio hints at a decrease in monolayer stability, which does not occur at the 30:1 ratio. The compressibility modulus analysis revealed that all three monolayers have similar in-plane elasticity behavior up to ∼ 20 mN m^–1^, differentiating at this point between the low and high melittin concentrations. The DOPE:DPPG and (DOPE:DPPG):MLT 30:1 monolayers show a similar trend, suggesting comparable stability and packing organization. In contrast, at a 10:1 ratio melittin disrupts the monolayer, increasing membrane fluidity and reducing structural organization, as demonstrated by the smaller maximum C_S_^–1^ values (Table 1).

**Figure 1:**
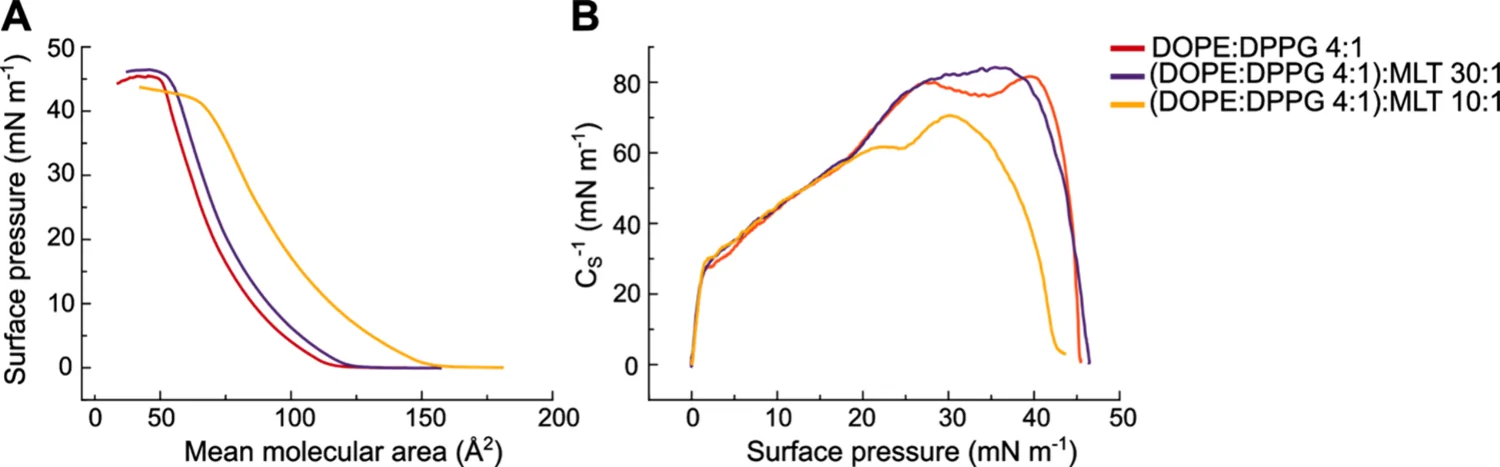
Interaction of melittin with lipid monolayers. (A) Surface pressure–area isotherms of Langmuir monolayers on 5 mM HEPES buffer subphase of DOPE:DPPG 4:1 (red), (DOPE:DPPG 4:1):MLT in ratios 30:1 (purple) and 10:1 (yellow). The area per molecule was calculated assuming that molecules of DOPE, DPPG, and melittin remain at the interface. (B) Compressional moduli of the mixed (DOPE:DPPG 4:1):MLT monolayers on the HEPES buffer subphase.

**Table 1:** Parameters for the Pure DOPE, DPPG, Melittin, and Mixed (DOPE:DPPG 4:1):MLT Monolayers on the HEPES Buffer Subphase

Composition	A_LO_ (Å^2^)^a^	A_30_ (Å^2^)^b^	Max C_S_^–1^ (mN m^–1^)^c^	π_col_ (mN m^–1^)^d^
DOPE	125	74	101	46
DPPG	138	48	174	65
DOPE:DPPG	118	62	82	45
(DOPE:DPPG):MLT 30:1	127	66	84	46
(DOPE:DPPG):MLT 10:1	157	82	71	43
MLT	-	175	48	37

a A_LO_ = lift-off area.

b A_30_ = area at 30 mN m^–1^.

c C_S_^–1^ = compressional modulus.

d π_col_ = collapse pressure.

The effect of melittin on the interfacial properties of the lipid monolayers was also studied using PM-IRRAS spectroscopy, providing insights into the molecular organization of the model membranes. Figure 2 presents the PM-IRRAS spectra of DPPG, DOPE, DOPE:DPPG, and the (DOPE:DPPG):MLT monolayers at a surface pressure of 30 mN m^–1^ in 5 mM HEPES buffer subphase. Besides the characteristic lipid vibrations, the spectra of the monolayers that contain the peptide display the amide I and amide II bands of melittin. The amide I region was further analyzed by spectral deconvolution (Figure S1). At both lipid-to-peptide ratios, the amide band is dominated by a component centered at 1658 cm^–1^, assigned to an α-helical conformation, indicating that melittin retains a predominantly α-helical shape upon membrane binding.^36,37^ The amide II band is observed at 1535 cm^–1^ for both peptide concentrations, a mode also associated with α-helical secondary structure.^38^ In addition, a higher-frequency amide I component, attributed to turns or terminal conformations, decreases in relative contribution with increasing melittin concentration, while the helical component increases, indicating reduced conformational heterogeneity. Since melittin is largely disordered in aqueous solution and folds into a predominantly α-helical conformation when binding to a membrane,^10,39,40^ the increased contribution of the α-helical component suggests a stronger membrane association at the higher peptide concentration.

**Figure 2:**
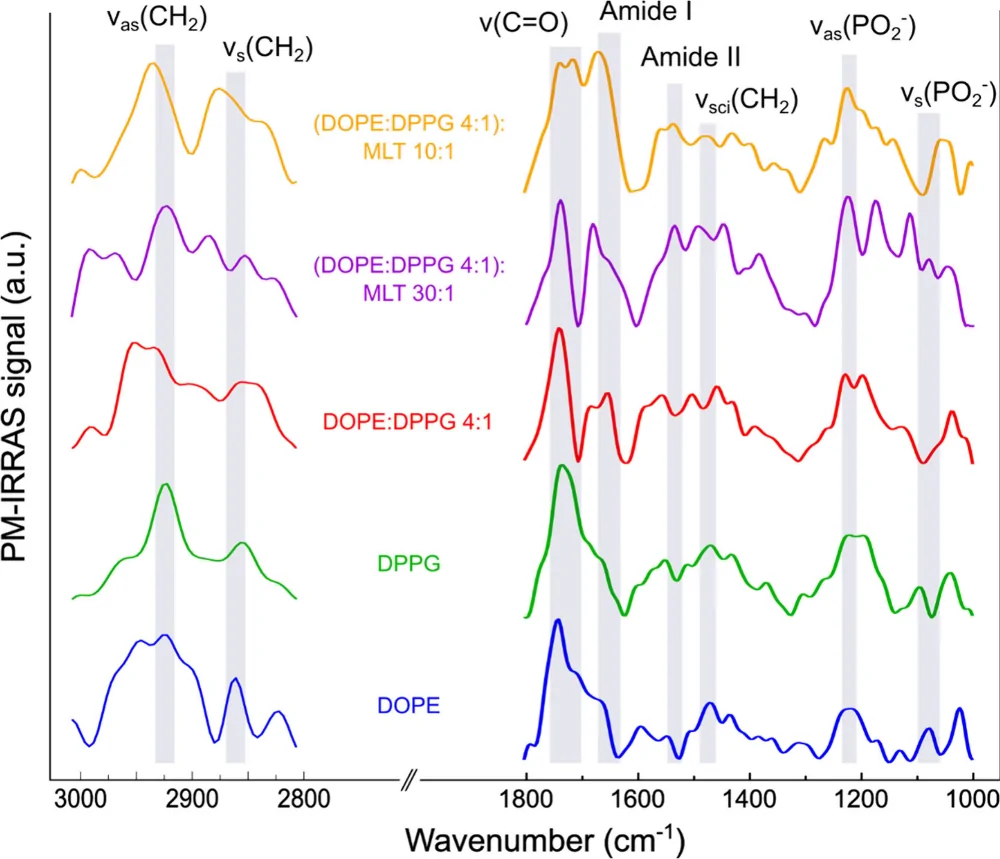
PM-IRRAS spectra of the DOPE (blue), DPPG (green), DOPE:DPPG 4:1 (red), (DOPE:DPPG 4:1):MLT 30:1 (purple), and 10:1 (yellow) L:P ratio Langmuir monolayers on a 5 mM HEPES subphase at a surface pressure of 30 mN m^–1^, in the 3000–2800 and 1800–1000 cm^–1^ ranges.

Lipid packing in the monolayers was examined by analyzing the asymmetric (*v*_*as*_) and symmetric (*v*_*s*_) stretching vibrations of methylene groups, which describe the conformational order of lipid alkyl chains. Highly ordered hydrocarbon chains show asymmetric and symmetric CH_2_ bands around 2920 and 2850 cm^–1^, respectively,^41^ with higher frequencies indicating decreased conformational order. The spectra in Figure 2 show that DOPE:DPPG monolayer has a more disordered hydrophobic core than DOPE and DPPG monolayers, as evidenced by an increase in the *v*_*as*_(CH_2_) frequencies from 2918 to 2928 cm^–1^ (Table S1 and Figure S2). The lipid mixing induces disorder in the acyl chains due to their different chain lengths and headgroups, increasing the fluidity of the monolayer,^42^ as confirmed by the decrease in the compressional modulus (Table 1). The presence of melittin at 30:1 L:P ratio slightly increases the acyl chains order, shifting the *v*_*as*_(CH_2_) stretching frequency down. In contrast, at a 10:1 ratio, the *v*_s_(CH_2_) band broadens and shifts to a higher frequency, indicating a decrease in the monolayer order,^43^ while the *v*_*as*_(CH_2_) frequency remains the same as the sample free of melittin. This change in CH_2_ frequencies, along with the changes in maximum *C*_*S*_^*–1*^ and collapse pressure values in Table 1, are the first indicators of melittin acting via different mechanisms depending on its concentration.

Another interesting spectral region in Figure 2 is between 1800–1000 cm^–1^, where the phosphate group signals appear between 1300 and 1000 cm^–1^ and the ester carbonyl band is seen around 1700 cm^–1^. These bands are sensitive to changes in the interfacial region of the monolayer: frequency variations indicate different hydrogen-bonding states of the functional groups and are therefore related to changes in headgroup hydration.^44^ All mixed monolayers show a band around 1740 cm^–1^, corresponding to non-hydrogen-bonded carbonyls buried in the hydrophobic region of the lipid arrangement. The 10:1 L:P monolayer shows additionally a second *v*(C=O) band around 1714 cm^–1^. This lower-frequency peak is associated with hydrogen bonding between the carbonyl groups and water molecules,^45,46^ and it is not observed for the sample with lower melittin concentration. The PO_2_^–^ stretching bands are highly sensitive to phosphate group hydration. The shift to lower frequencies of the *v*_as_(PO_2_^–^) band from 1227 cm^–1^ in DOPE:DPPG to 1224 cm^–1^ at the 10:1 L:P ratio, along with the shift of the *v*_s_(PO_2_^–^) band from 1065 to 1060 cm^–1^, indicates a higher degree of hydration of the phosphate group. This suggests that the interaction with melittin at this concentration changes the lipid headgroup environment, facilitating water penetration around the phosphate and carbonyl groups.^44^ In contrast, at a 30:1 ratio the *v*_as_(PO_2_^–^) band remains unchanged while the *v*_s_(PO_2_^–^) increases significantly from 1065 cm^–1^ to 1080 cm^–1^. Because the asymmetric PO_2_^–^ stretching mode is more sensitive to changes in hydration and local electric fields than the symmetric mode, the selective upshift in *v*_s_(PO_2_^–^) suggests that interaction with the melittin induces conformational or orientation changes on the phosphate group, such as a reduction in the motional freedom of the polar headgroups.^47,48^ The upshift is also consistent with dehydration of the phosphate groups, possibly due to competition between peptide and water molecules for bonding sites.

### 3.2 Melittin-Induced Morphological Changes at the Microscopic Scale

BAM was used to capture morphological changes of the monolayers. Figure S3 shows BAM images taken at various surface pressures for the three Langmuir monolayers. In the absence of melittin the lipid mixture forms a film with bead-like domains, which become more noticeable near monolayer collapse. These brighter spots in Figure S3 correspond to condensed microdomains where the saturated lipids interact more strongly while coexisting within the LE phase favored by the predominant unsaturated DOPE composition. The general morphology remains unchanged with melittin incorporation, consistent with the surface pressure–area results, as all samples exist in the liquid-expanded phase.^49,50^ However, at a 30:1 L:P ratio, the bead-like domains appear earlier (i.e., at a lower surface pressure) and in higher numbers than they do for DOPE:DPPG and the 10:1 L:P, indicating a slight increase in lateral rigidity. This observation is in accordance with the very small increase in *C*_*S*_^*–1*^ described previously and the increase in alkyl chain order observed by PM-IRRAS. In the case of the 10:1 L:P monolayer, the domains first appear at higher pressures than for the other two and seem to be flattened and less contrasting, giving the monolayer a more uniform appearance. These features align with the increased acyl chains disorder and in-plane elasticity revealed by the compressibility modulus changes. At this higher concentration melittin seems to act as a fluidizing agent, as higher compression is required to induce lateral homogeneities that form easier at a lower melittin concentration.^47^

AFM was used to further investigate the surface topography and nanoscale structural features of lipid thin films, providing information about film thickness and lateral heterogeneity that complements the insights from BAM. In addition to height imaging, AFM phase imaging was employed to probe local variations in viscoelastic and adhesive properties of the films.^51^ Although BAM and AFM probe different systems, their combined use provides a more detailed interpretation of melittin-lipid interactions under varied conditions. The AFM measurements were performed on dry spin-coated multilayer films and therefore provide complementary evidence of peptide-induced structural reorganization on the lipid film rather than a direct readout of membrane insertion or permeabilization mechanisms.^51^ Figure 3 shows that the lipid film thickness decreases drastically with increasing melittin concentration.^52^ Since under the equal conditions the thickness of the film prepared by a spin-coating technique depends on the material viscosity, thinning of the film indicates the viscosity reduction after melittin addition. This supports the conclusion on the effect of melittin on lipid–lipid interactions observed in Langmuir monolayers, where higher melittin concentrations cause a decrease in intermolecular interactions resulting in the decrease of the compressibility modulus and collapse pressure. Although these are different model systems, the AFM results indicate that melittin alters the structural organization or lipid films, even in dry multilayer stacks.

**Figure 3:**
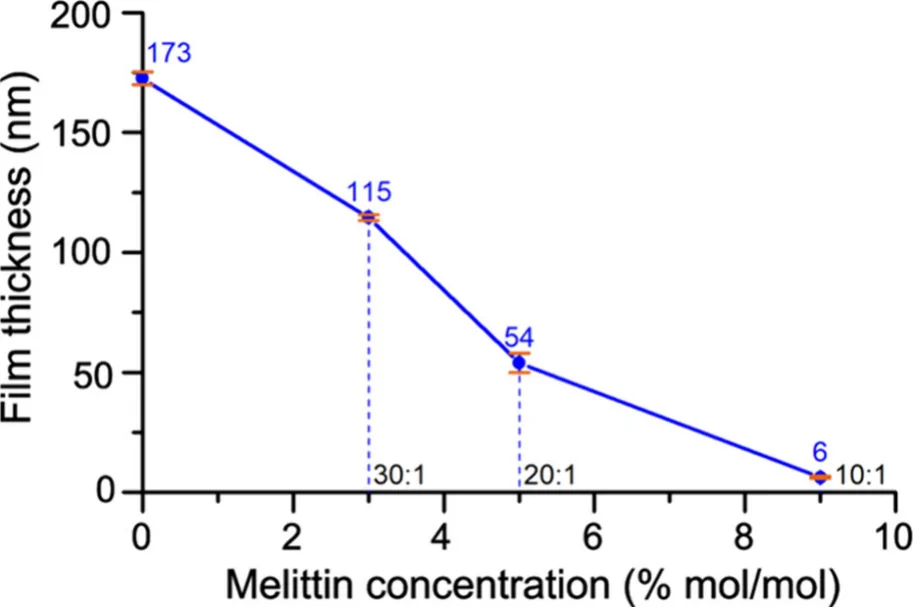
Thickness of spin-coated lipid films. Film thickness (determined from a scratch experiment) as a function of the molar concentration of melittin in the precursory solution. The equivalent L:P ratios are indicated at each concentration point; all measurements were done under low vacuum environment.

The effect of melittin is also evidenced in the topographical and phase images of the film (Figures 4 and S2). In the absence of melittin, the surface morphology matches what is expected from a thick phospholipid dry film,^53^ with multiple stacked lipid bilayers forming a heterogeneous surface with significant height variations. As melittin concentration increases, the film undergoes progressive reorganization, reducing the lateral inhomogeneities. This change is observed as the fusion and homogenization of what were originally separate “stacks”, evidenced in Figure 4. The images for the 30:1 L:P and especially the 20:1 L:P ratio resemble reported ones for intermediate stages of a temperature-induced phase transition.^54,55^ This suggests that increasing melittin content could promote a structural organization of the dry lipid film that resembles the intermediate stage of a gel-to-liquid-crystalline phase transition reported for hydrated lipid systems.^56^ Moreover, at the highest melittin content, the average film thickness is in the range of 4 to 8 nm, similar to a single hydrated lipid bilayer,^57^ showing that the peptide is less effective at perturbing a continuous layer supported on a solid substrate, most likely due to stabilizing adhesion forces. This observation provides further evidence of the ability of melittin to reorganize dry lipid multilayered stacks prepared by codeposition with the peptide. Because the present AFM was performed on dry codeposited multilayer films rather than supported lipid bilayers in buffer, it cannot directly assess peptide partitioning, insertion, or pore formation in hydrated membranes. Such a configuration would provide more extensive information on a more biologically relevant model.

**Figure 4:**
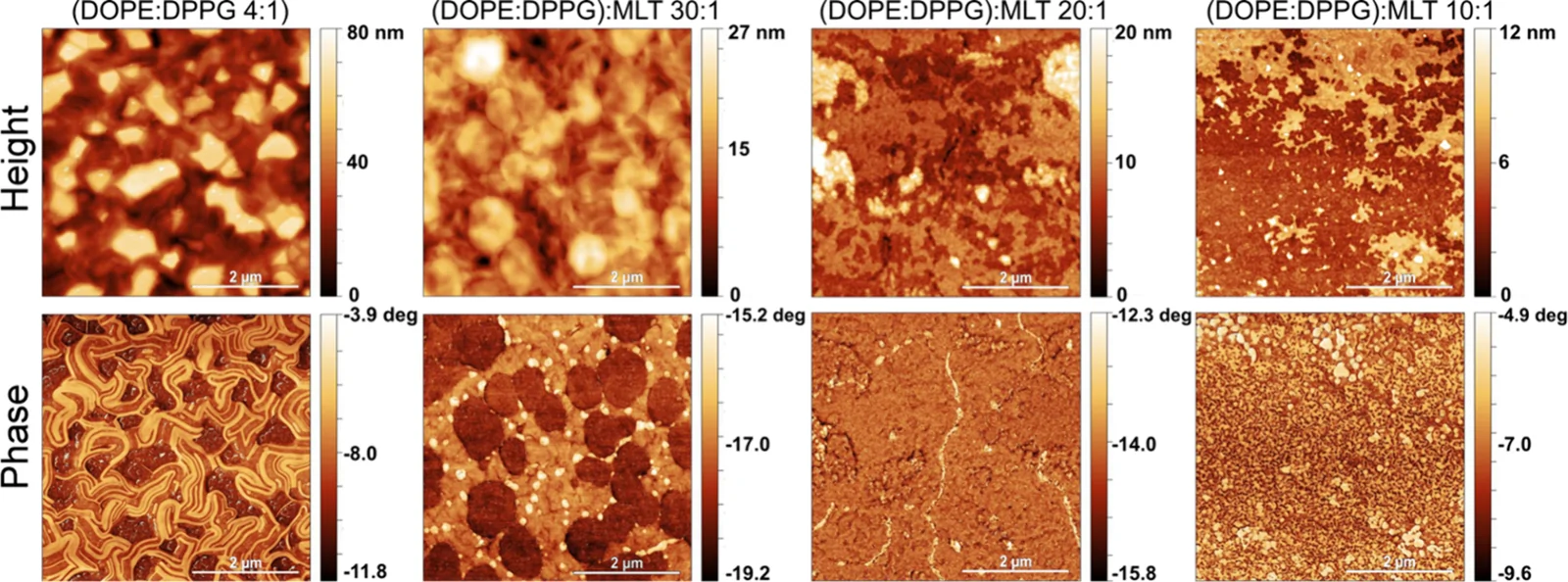
Effect of melittin on the organization of lipid films. AFM topographical and phase images of spin-coated lipid films of DOPE:DPPG 4:1, (DOPE:DPPG 4:1):MLT 30:1, 20:1, and 10:1. Each subset corresponds to a 5 × 5 μm scan area; scale bar is 2 μm; all measurements were done under low vacuum environment.

AFM phase imaging is done simultaneously with topography imaging in the noncontact mode, and it allows us to resolve features in soft samples that would otherwise be hidden. In the case of lipid samples, it can help identify compositional heterogeneities on the same surface that arise from different lipid domains or the presence of proteins or peptides.^58,59^ For the DOPE:DPPG sample, the film exhibits the coexistence of gel–fluid domains, characteristic of a binary lipid mixture with distinct phase transition temperatures.^60^ This behavior is perturbed by the melittin at lower concentrations and completely disrupted at higher concentrations. The phase image for the 30:1 L:P film in Figure 4 shows that the system retains some of the original phase organization, similar to the peptide-free sample. However, when the peptide concentration is higher, the original compositional organization is severely disrupted. Furthermore, at the highest peptide concentration, small aggregates are also identified in the phase image that were not observed at lower concentrations. Hence, low melittin concentrations can cause localized phase disturbances, as observed in BAM images while simultaneously affecting the large-scale organization of the dry lipid film. Similar effects have been reported for pore-forming toxins from bacteria.^60^

### 3.3 Specificity of Melittin Binding and Molecular Interactions

To further investigate melittin’s interaction with phospholipid membranes, we employed multilamellar lipid vesicles (MLVs) as a model system. These vesicles were prepared in the same ratios as for the other membrane models to mimic the multilayered structure of cellular membrane in an aqueous environment. Using DLS and ssNMR we can correlate large-scale hydrodynamic changes with molecular-level interactions. DLS analysis (Table S2 and Figure S5) showed that the DOPE:DPPG MLVs have a predominant population with an average hydrodynamic diameter of 890.9 ± 498.7 nm, with minor contributions from additional populations indicating some heterogeneity. For the 30:1 L:P ratio, the vesicle size distribution remains largely unimodal, with the major peak accounting for 86% of the scattering intensity and a reduced average of 687.1 ± 311.8 nm, while the 10:1 L:P MLVs exhibit a pronounced decrease in the dominant hydrodynamic diameter to 331.1 ± 105.1 nm. The progressive narrowing of the main population indicates increasing uniformity in vesicle size with increasing melittin concentration. The pronounced reduction of the hydrodynamic diameter with increasing melittin content is consistent with the lipid reorganization caused by melittin in the films studied by AFM.

SsNMR spectroscopy of ^1^H, ^13^C, and ^31^P was used to investigate the effect of melittin on the MLVs at an atomic level. For the sake of comparison, all ^1^H and ^13^C chemical shifts (δ^1^H and δ^13^C) are listed in Table S4. Comparison of the ^1^H spectra for MLVs with and without melittin reveals the most pronounced ^1^H chemical shift changes (Δδ^1^H) for resonances associated with the phospholipid headgroups, glycerol necks, and the atoms C2 and C3 (Figure 5). Analysis of this data suggests that melittin exhibits a stronger interaction with the polar regions than with other parts of the phospholipid molecule. The ^13^C spectra further confirm that melittin primarily interacts with the glycerol ester region and the DPPG headgroups of the phospholipids (Figure 6). The chemical shift change (Δδ) plot in Figure 6B shows that the most significant Δδ^13^C occur for the C2 and C3 positions, the g2 carbon, and the DPPG headgroup carbons, with all affected resonances following a similar trend for both melittin concentrations. Another important observation is the pronounced Δδ^13^C of the carbonyl signal (Figure 6A, inset), along with the appearance of a second component for the 10:1 L:P MLVs, indicating the presence of distinct local environments. In all cases, the magnitude of the Δδ^13^C is larger at the 10:1 L:P ratio, indicating a stronger perturbation of the bilayer at higher melittin concentration.

**Figure 5:**
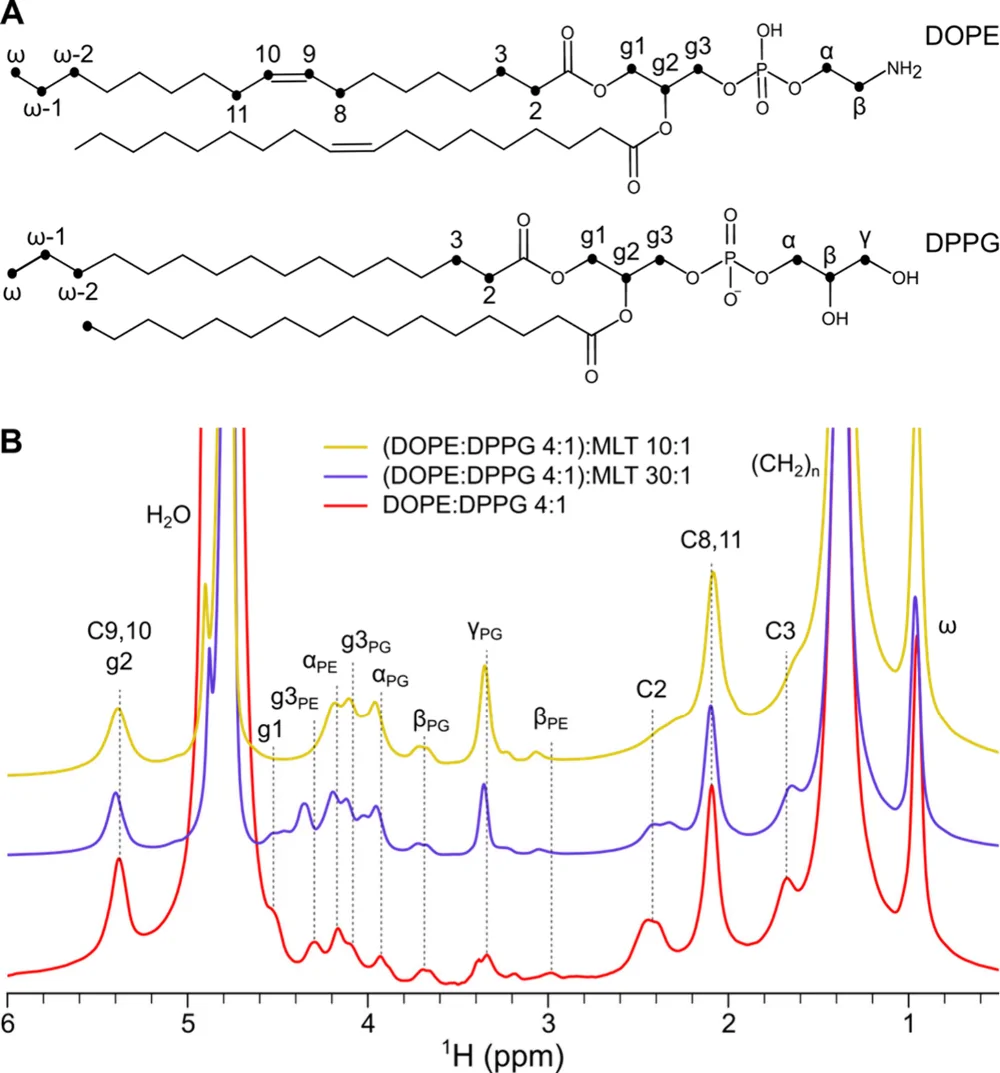
Interaction of melittin with complex MLVs studied by ^1^H ssNMR. (A) Structures of DOPE and DPPG phospholipids with atomic assignment. (B) ^1^H MAS spectra of DOPE:DPPG 4:1, (DOPE:DPPG 4:1):MLT 30:1, and 10:1 L:P ratio MLVs. All spectra were acquired at 300 K on a 9.4 T spectrometer using a MAS speed of 10 kHz.

**Figure 6:**
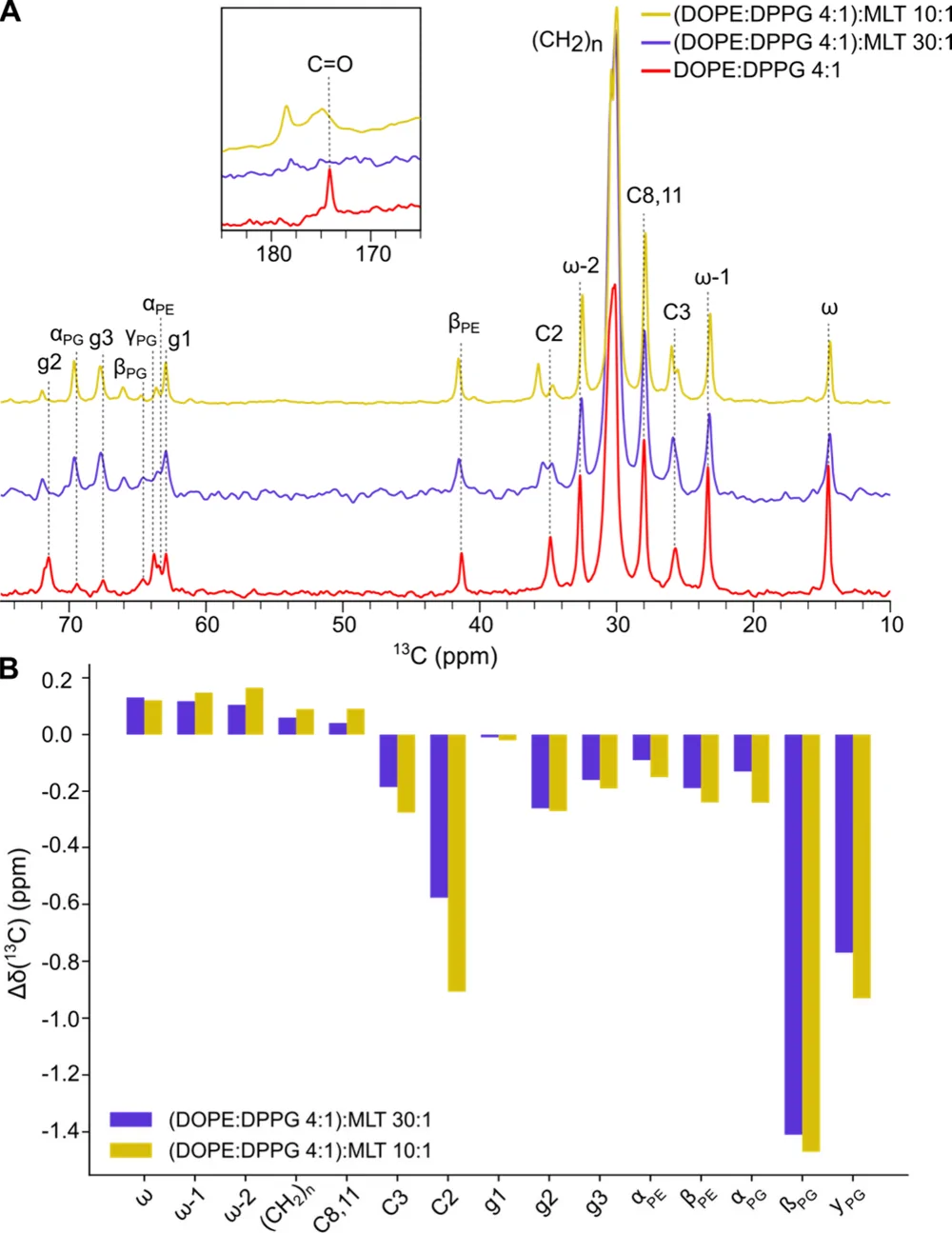
Effect of melittin on complex MLVs studied by ^13^C ssNMR. (A) ^13^C INEPT spectra of DOPE:DPPG 4:1, (DOPE:DPPG 4:1):MLT 30:1, and 10:1 L:P ratio MLVs**;** the inset shows the carbonyl region from the direct excitation spectra. (B) Plot showing the relevant changes in the MLVs ^13^C chemical shifts (Δδ^13^C) caused by their interaction with melittin. All spectra were acquired at 300 K on a 9.4 T spectrometer under a MAS speed of 10 kHz.

The ^31^P direct excitation spectra in Figure 7 reveal noticeable changes in both intensity and band shape upon peptide addition. Deconvolution of the signals was carried out for all samples, and in every case the resulting components were consistent with the composition of the prepared MLVs (80% DOPE and 20% DPPG). For DOPE:DPPG MLVs without melittin, the broad band arises from the overlap of the two phosphorus signals, with the DOPE contribution centered at −0.02 ppm and the DPPG contribution at 0.30 ppm. Upon addition of melittin at both concentrations, the spectra become asymmetric, and a distinct shoulder appears at higher chemical shift. For MLVs at both the 30:1 and the 10:1 L:P ratios, the DOPE and the DPPG signals move to higher δ^31^P; however, the magnitude of this change is more pronounced for DPPG. This observation is consistent with ^13^C results, which indicate a stronger interaction of the peptide with PG headgroups compared to PE. This conclusion is further supported by ^31^P T_1_ results, which show that at a 30:1 L:P ratio the interaction with melittin leads to a reduction in the mobility of the DPPG phosphate group, evidenced by an increase of its T_1_. Previous works have shown that an increase in the phosphorus T_1_ is commonly associated with increased lipid rigidity, reflecting a more restricted motion of the phosphate group relative to the glycerol backbone.^55,56^ At a ratio of 10:1 L:P this effect becomes slightly less pronounced, although the T_1_ value remains higher than for the melittin-free MLVs, indicating a persistently increased rigidity. In contrast, the T_1_ for DOPE decreases, although the magnitude of this change is substantially smaller than that observed for DPPG. The T_1_ change also remains consistent across both melittin concentrations, indicating that melittin causes a comparatively weaker perturbation of the DOPE phosphate environment.

**Figure 7:**
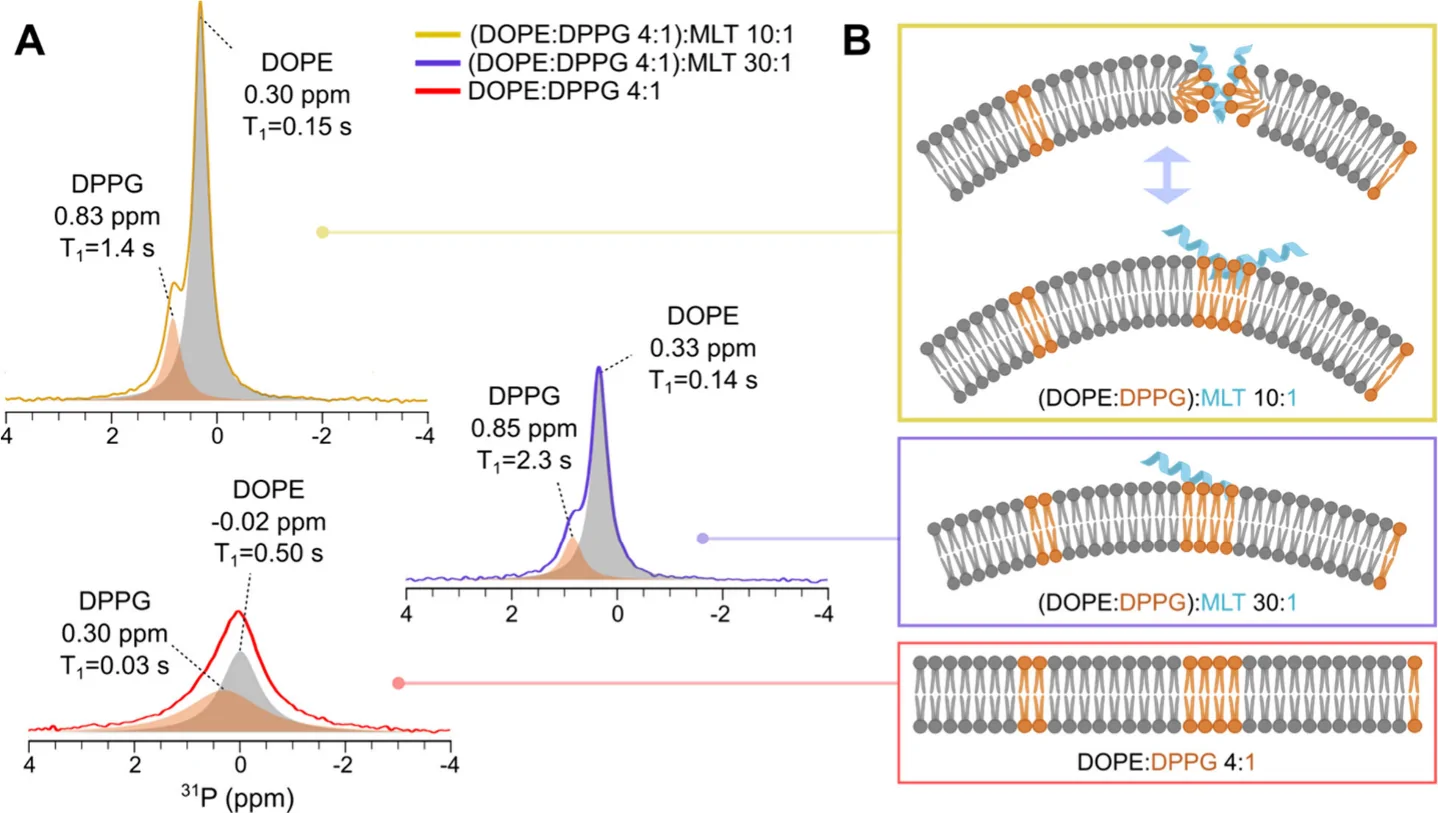
Melittin’s preferential interaction with DPPG. (A) ^31^P direct excitation spectra of the DOPE:DPPG 4:1, (DOPE:DPPG 4:1):MLT 30:1, and 10:1 L:P ratio MLVs. Each subset shows the chemical shift and T_1_ values of the deconvoluted components assigned as DOPE and DPPG. (B) Pictogram description of the preferential interaction of melittin with DPPG at different melittin concentrations. All spectra were acquired at 300 K on a 9.4 T spectrometer using a MAS speed of 10 kHz.

### 3.4 Computational Insight into Melittin-Lipid Interactions

Molecular dynamics simulations were carried out on three models: a DOPE:DPPG (4:1) lipid bilayer (system I), the same bilayer with two melittin chains (system II), and with four melittin chains (system III) (Figure S6). These simulations were intended to capture trends in bilayer behavior as a function of the peptide content. While direct comparison between *in silico* models and experimental systems probed by different techniques is not straightforward, the simulations provide additional insight into the observed melittin-lipid interactions. Table 2 summarizes the MD results of the bilayer structural parameters for each system. Consistent changes are observed in membrane fluidity, bilayer thickness, area per lipid, and overall bilayer organization with an increasing melittin content. The increase in membrane fluidity, reflected in the diffusion coefficient trend, indicated a progressive fluidization of lipid packing as peptide concentration increases.^61,62^ A gradual decrease in the bilayer thickness is also observed as the melittin concentration increases. This disruptive behavior is consistent with the AFM measurements showing the effect of melittin on lipid organization (see Figures 3 and 4) at higher melittin content, as well as DLS results indicating a decrease in hydrodynamic diameter.

**Table 2:** Comparison of Structural Parameters of Three Lipid Bilayers with Zero, 2, and 4 Melittin Peptides, Obtained by MD Simulations^a^

		Area per lipid (Å^2^)				
System	System description	Total lipids	DOPE	DPPG	Membrane thickness (Å)	Diffusion coefficient (×10^–7^ cm^2^/s)
I	Lipid bilayer	73. 01 ± 1.46	72.51 ± 1.04	75.00 ± 1.14	38.37 ± 1.13	3.22 ± 0.18
II	Lipid bilayer +2 MLT	73. 81 ± 6.61	72.73 ± 4.12	78.13 ± 11.27	37.76 ± 1.09	3.42 ± 0.19
III	Lipid bilayer +4 MLT	74.68 ± 8.93	73.59 ± 7.96	79.06 ± 11.02	37.14 ± 1.07	3.64 ± 0.21

a 200 ns of sampling.

Another significant finding is the increase in the area per lipid, with a stronger effect observed for DPPG compared to DOPE. For DPPG, the area per lipid increases by approximately 4 Å^2^ from system I to system III, whereas DOPE exhibits a smaller increase of about 1 Å^2^. This trend in area per lipid obtained from the bilayer simulations mirrors the one observed in Langmuir monolayers, where increases of 4 Å^2^ and 20 Å^2^ were measured for the 30:1 and 10:1 L:P ratios, respectively (see Figure 1 and Table 1). Furthermore, the standard deviation of the area per lipid increases more drastically for DPPG, indicating that a higher sensitivity of this lipid to the presence of melittin; this preferential perturbation of DPPG is in agreement with the ssNMR observations. The two-dimensional projections of the area per lipid evolution over the simulation time shown in Figure 8 reveal that the largest increases coincide with melittin binding sites, consistent with localized lipid reorganization driven by peptide association. Increased membrane undulations were observed with higher melittin content, as shown in the snapshots in Figure 8. This structural phenomenon can also be pointed out in the density plots of the DOPE glycerol ester group, where the internal leaflet profile deviates from a standard Gaussian distribution and shifts toward the bilayer center (Figure S7).

**Figure 8:**
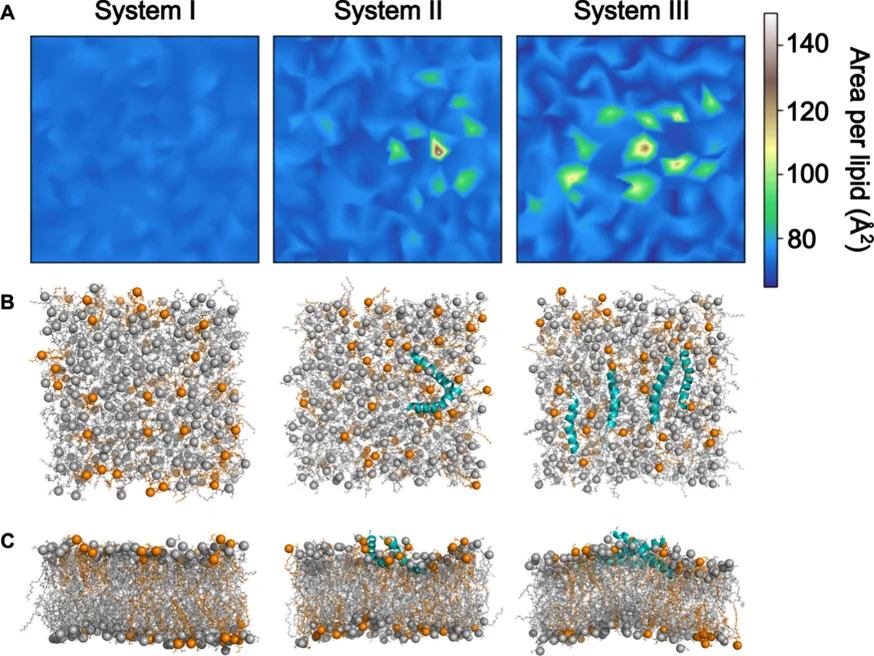
Lipid bilayer perturbation with increasing melittin content. (A) Projections of the area per lipid onto the xy plane for systems I, II, and III. The areas per lipid were averaged over 200 ns of sampling based on the center of mass of each lipid. Snapshots from the (B) top and (C) transversal view at 150 ns of the MD runs for systems II and III. DOPE and DPPG phospholipids are shown in gray and orange, respectively. The P atoms shown are spheres. Water molecules and ions are omitted for clarity.

Figure 9 shows the acyl chain order parameters for all three systems, depicting the decrease in the order for all atoms with an increasing melittin content. However, the magnitude of these changes is not sufficient to assume significant disruption of the bilayer hydrophobic center, consistent with ssNMR results and the unchanged chemical shifts observed for the signals corresponding to the acyl chains.

**Figure 9:**
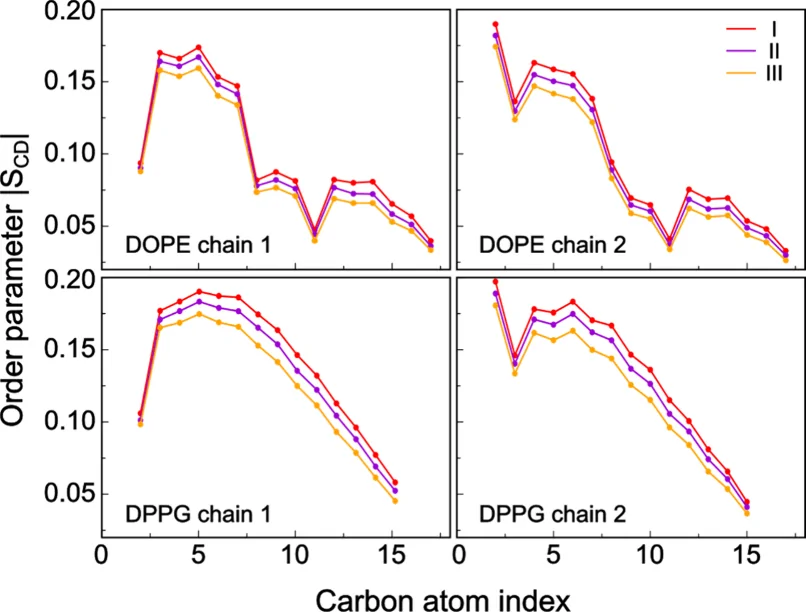
Order parameters |S_CD_| of systems I (red), II (purple), and III (orange) for both chains of DOPE and DPPG at 323.15 K as a function of the carbon atom indexes (as assigned in Figure 5A). The order parameters were averaged over 200 ns of sampling.

### 3.5 Integrated Mechanistic Description

We combined complementary experimental techniques and molecular dynamics simulations to study how melittin interacts with anionic membranes comprising DOPE:DPPG (4:1). By using membrane models that span different length scales and hydration states, we connect interfacial behavior, membrane morphology, and molecular-level dynamics to peptide concentration and, ultimately, to the melittin mechanism of action. At low peptide concentration (30:1 L:P), melittin associates with the membrane without inducing major structural changes. Langmuir monolayers remain in the liquid-expanded phase, showing only a slight expansion and minimal changes in compressibility. BAM reveals morphologies very similar to those of peptide-free membranes, with only subtle changes in lateral organization. AFM imaging of spin-coated lipid films complements the BAM results by showing that while there is some disturbance of the morphology caused by the introduction of melittin at low concentration, the film appears to retain an overall similar lateral organization to that of the peptide-free film. Spectroscopic measurements indicate that melittin primarily interacts with the interfacial region: PM-IRRAS and ssNMR show preferential interaction with DPPG headgroups and reduced molecular motion of phosphate groups without evidence of increased hydration or deep bilayer penetration. In particular, PM-IRRAS reveals a slight increase in order in the lower acyl chains, a pattern that has been associated with the carpet model, where peptides bind parallel to the outer leaflet and induce compensatory ordering deeper in the bilayer.^63^ DLS and MD simulations further support this picture, showing mild thinning, modest vesicle size reduction, and localized lipid rearrangements near melittin binding sites, while the overall membrane continuity is preserved. The increased constraint of the phosphate groups and the stronger interaction with the interfacial region indicate that, at this ratio, melittin adsorbs parallel to the membrane surface and causes nonspecific permeabilization without stable pore formation, consistent with a carpet-like mechanism^64^ (as represented in Figure 7A).

At higher peptide concentration (10:1 L:P), the nature of the interaction changes. Langmuir monolayers exhibit stronger expansion, reduced collapse pressure, and increased acyl chain disorder, indicating enhanced membrane fluidity. BAM shows delayed and flattened lateral heterogeneities, while AFM reveals the spin-coated films were much thinner and melittin seemed to cause lateral homogenization of the lipid films. PM-IRRAS detects increased hydration of carbonyl and phosphate groups and an increase in the α-helical conformation of melittin, suggesting water penetration into the interfacial region and a stronger binding of the peptide to the membrane. Additionally, the analysis of CH_2_ stretching signals shows that the increase in melittin concentration leads to a different type of interaction with the monolayers and causes a slight increase in disorder of the acyl chain region. ^13^C and ^31^P ssNMR reveals larger chemical shift changes and multiple local environments, particularly involving DPPG, while MD simulations are consistent with the experimental observations, showing increased lipid diffusion, larger area per lipid, enhanced bilayer undulations, and reduced acyl chain order. These combined features consistently indicate that increasing melittin concentration leads to a different mode of membrane interaction which causes greater perturbation. The toroidal-pore model has been long associated with melittin mechanisms of action, and it could explain the results here obtained; however, the absence of strong acyl chain chemical shift perturbations in ssNMR (Figures 5 and 6) suggests that, rather than long-lived pores, melittin may induce highly dynamic and short-lived defects. This behavior is consistent with proposed kinetic variants of the carpet model, in which peptide adsorption continues until a transient pore forms, allowing peptide translocation across the bilayer before the membrane reseals.^65,66^ In this context, the increased hydration detected by PM-IRRAS and the marked increase in membrane fluidity would facilitate transient water penetration, while the lack of persistent acyl chain perturbations reflects the short lifetime of these pore-like states (Figure 7B). In our system, this scenario is further supported by DLS results, which show a much larger decrease in MLV size at higher melittin concentration, consistent with enhanced membrane remodeling and vesicle reorganization following peptide translocation. While the formation of a transient pore cannot be directly observed with the techniques here implemented, the observations here presented would support that interpretation. This is also consistent with recent results in which melittin has been described as acting through transient, dynamic defects or toroidal-pore intermediates at high concentrations that could initiate from states aligned to the membrane surface.^12,66^

The analysis of ^31^P spin–lattice relaxation times (T_1_) provides insight on the peptide binding to the membrane. The molecular motion that dominates phosphate T_1_ relaxation does not correspond to the motion of the lipid molecule as a whole but, rather, to reorientation of the phosphate group relative to the glycerol backbone.^67^ This distinction is crucial, as it shows that melittin strongly constrains the motion of the phosphate headgroup through specific interactions at the membrane interface, without necessarily restricting global lipid mobility. To our knowledge, this type of relaxation-based analysis of peptide–lipid interactions has not been applied to antimicrobial peptides, and it provides a sensitive probe of headgroup-level dynamics that complements chemical shift and structural measurements.

## 4 Conclusions

Taken together, the results here presented indicate that melittin acts via a concentration-dependent mechanism in DOPE:DPPG (4:1) membranes, a behavior that has been observed as well for membranes of different compositions.^11,39,68,69^ At low peptide loading, melittin remains surface bound. It perturbs lipid order asymmetrically across the bilayer and induces nonspecific permeabilization consistent with the carpet model. At higher concentrations, increased hydration, fluidity, and membrane remodeling are consistent with greater membrane perturbation, which may reflect transient, dynamic defects as proposed kinetic-carpet or transient toroidal-pore models. Across all techniques DPPG is more strongly affected than DOPE, consistent with what the literature reports and highlighting melittin selectivity toward anionic membranes. More broadly, the mechanism of action of antimicrobial peptides must be evaluated on a case-by-case basis, as it depends not only on the peptide but also on membrane composition, including headgroup chemistry, acyl chain length, degree of unsaturation, and lipid complexity. For the DOPE:DPPG (4:1) system studied here, the combination of interfacial, spectroscopic, microscopic, and computational techniques provides a coherent, multiscale picture of melittin–membrane interactions. This integrated approach offers a robust framework that can be extended to other antimicrobial peptides and membrane systems, where different compositions may lead to varying mechanistic outcomes.

## Supplementary Material



## Notes

PM-IRRAS frequencies for all Langmuir monolayers; size distributions by intensity of MLVs obtained by DLS; ^1^H, ^13^C and ^31^P chemical shifts for all samples; Deconvolution of PM-IRRAS amide I and acyl chain spectral regions; Brewster angle microscopy images of the Langmuir monolayers; extended AFM topography and phase imaging; snapshots of initial steps of MD runs for all three; density profiles of peptide, headgroups and glycerol ester groups after MD runs for all three systems (PDF)

## Abbreviations

AMP: antimicrobial peptide
MLT: melittin
L:P: lipid-to-peptide ratio
MLVs: multilamellar vesicles
DLS: Dynamic light scattering
AFM: Atomic force microscopy
PM-IRRAS: Polarization-modulation infrared reflection–absorption spectroscopy
BAM: Brewster angle microscopy
ssNMR: solid-state nuclear magnetic resonance
MD: molecular dynamics
