# Supplementary material for: Concentration-Dependent Modes of Melittin-Induced Membrane Destabilization: An Integration of Experimental and Computational Tools
Source: Langmuir. 2026 Sep 9;42(37):27214–25. doi: 10.1021/acs.langmuir.6c02887 (PMC13602908; doi:10.1021/acs.langmuir.6c02887)
Supplement: Supplementary file 1 [file la6c02887_si_001.pdf]

Supporting Information

Concentration-dependent modes of melittin-induced membrane destabilization: an integration of experimental and computational tools

Andrea Chacón Calderón[1], Pavel Zelenovskii[2], Marcio Soares[1], Ellen C. Wrobel[3], Osvaldo N. Oliveira Jr.[3], Daniel Pereira[1], Mariana Sardo[1], Luís Mafra[1], Ana Barros-Timmons[1*], Ildefonso Marín-Montesinos[1]*.

[1]Department of Chemistry & CICECO−Aveiro Institute of Materials, University of Aveiro, 3810-193 Aveiro, Portugal.
[2]Department of Physics & CICECO−Aveiro Institute of Materials, University of Aveiro, Aveiro 3810-193, Portugal.
[3] Sao Carlos Institute of Physics, University of Sao Paulo, CP 369, 13560-970 São Carlos, SP, Brazil

*Corresponding authors: anabarros@ua.pt and imarin@ua.pt

Contents

Table S1. PM-IRRAS frequencies of the pure lipids, DOPE:DPPG 4:1 and (DOPE:DPPG 4:1):MLT monolayers on 5 mM HEPES buffer subphase at a surface pressure of 30 mN m^{-1}.
Table S2. Size and size distribution by intensity obtained by dynamic light scattering for MLVs of DOPE:DPPG 4:1, (DOPE:DPPG 4:1):MLT 30:1 and 10:1 ratio.
Table S3. ^{1}H and ^{13}C chemical shifts changes for MLVs of DOPE:DPPG 4:1, (DOPE:DPPG 4:1):MLT 30:1 and 10:1 ratio.
Table S4. ^{31}P chemical shifts for the deconvoluted spectra and T_1 relaxation times for MLVs of DOPE:DPPG 4:1, (DOPE:DPPG 4:1):MLT 30:1 and 10:1 ratio.

Figure S1. Study of melittin secondary structure through the amide I band in PM-IRRAS.
Figure S2. Effect of melittin on acyl chain region of Langmuir monolayers observed by PM-IRRAS.
Figure S3. Effect of melittin on Langmuir monolayers observed by Brewster angle microscopy.
Figure S4. Interaction of melittin with thin lipid films observed by AFM topographical and phase images of the lipid films.
Figure S5. Size distribution changes induced in MLVs by melittin.
Figure S6. Snapshots from the top view of the initial step (1 ns) of the MD runs for systems I, II, and III.
Figure S. Density profiles for systems I, II and III averaged over 200 ns of sampling.

Table S1. PM-IRRAS frequencies of the pure lipids, DOPE:DPPG 4:1 and (DOPE:DPPG 4:1):MLT monolayers on 5 mM HEPES buffer subphase at a surface pressure of 30 mN m^{-1}.

Composition	PM-IRRAS signal wavenumber (cm^{-1})					
	$\nu_{as}(CH_2)$	$\nu_s(CH_2)$	$\nu(C{=}O)$	$\nu_{sci}(CH_2)$	$\nu_{as}(PO_2^-)$	$\nu_s(PO_2^-)$
DOPE	2918	2856	1740	1470	1218	1076
DPPG	2918	2849	1732	1468	1220	1094
DOPE:DPPG 8:2	2927	2846	1739	1458	1227	1065
(DOPE:DPPG):MLT 30:1	2917	2846	1740	-	1227	1080
(DOPE:DPPG):MLT 10:1	2928	2856	1738<br>1714	1477	1224	1060

Table S2. Size distribution by intensity obtained by dynamic light scattering for MLVs of DOPE:DPPG 4:1, (DOPE:DPPG 4:1):MLT 30:1 and 10:1 ratio. The intensity percentage of each population is shown inside the parenthesis.

PDI / Peak information	DOPE:DPPG	(DOPE:DPPG):MLT 30:1	(DOPE:DPPG):MLT 10:1
PDI	0.756	0.539	0.628
Peak 1 (%)	890.9 ± 498.7 nm (73.9)	687.1 ± 311.8 nm (85.6)	333.1 ± 105.1 nm(79.3)
Peak 2 (%)	126.4 ± 43.11 nm (19.8)	103.6 ± 37.05 nm (12.2)	63.12 ± 16.01 nm (12.5)
Peak 3 (%)	4706 ± 824.3 nm (6.3)	5047 ± 584.3 nm (1.9)	4677 ± 880.3 nm (8.2)

Table S3. ^{1}H and ^{13}C chemical shifts changes for DOPE:DPPG 4:1, (DOPE:DPPG 4:1):MLT 30:1 and 10:1 ratio MLVs. All spectra were acquired at 300 K on a 9.4 T spectrometer using a MAS speed of 10 kHz.

Signal	^{1}H chemical shift (ppm)			Signal	^{13}C chemical shift (ppm)		
	DOPE:DPPG	(DOPE:DPPG): MLT 30:1	(DOPE:DPPG): MLT 10:1		DOPE:DPPG	(DOPE:DPPG): MLT 30:1	(DOPE:DPPG): MLT 10:1
ω	0.95	0.96	0.96	ω	14.53	14.4	14.41
$(CH_2)_n$	1.36	1.37	1.36	ω-1	23.33	23.21	23.18
C8, 11	2.10	2.10	2.09	ω-2	32.68	32.57	32.51
C9, 10	5.38	5.40	5.39	$(CH_2)_n$	30.14	30.08	30.05
C3	1.68	1.65	1.63	C8, 11	27.99	27.95	27.9
C2	2.40	2.33	2.30	C3	25.71	25.89	25.98
	2.45	2.42	2.39	C2	34.85	35.43	35.76
g1	4.53	4.50	-	g1	62.93	62.94	62.95
g2	-	5.08	5.08	g2	71.7	71.96	71.97
g3-PE	4.30	4.36	-	g3	67.53	67.69	67.72
g3-PG	4.10	4.12	4.11	α_{PE}	63.47	63.56	63.62
α_{PE}	4.17	4.20	4.19	β_{PE}	41.34	41.53	41.58
β_{PE}	2.99	3.05	3.07	α_{PG}	69.4	69.53	69.64
α_{PG}	3.93	3.96	3.96	β_{PG}	64.61	66.02	66.08
β_{PG}	3.68	3.70	3.69	y_{PG}	63.81	64.58	64.74
y_{PG}	3.34	3.36	3.35				

Table S4. ^{31}P chemical shift changes ($\Delta\delta^{31}P$) for the deconvoluted spectra and T_1 relaxation times for the DOPE:DPPG 4:1, (DOPE:DPPG 4:1):MLT 30:1 and 10:1 ratio MLVs. All spectra were acquired at 300 K on a 9.4 T spectrometer using a MAS speed of 10 kHz.

Signal	$\Delta\delta(^{31}P)$ (ppm)		^{31}P relaxation time T_1 (s)		
	(DOPE:DPPG):MLT 30:1	(DOPE:DPPG):MLT 10:1	DOPE:DPPG	(DOPE:DPPG):MLT 30:1	DOPE:DPPG):MLT 10:1
DOPE	0.35	0.32	0.50 ± 0.05	0.14 ± 0.01	0.15 ± 0.01
DPPG	0.56	0.53	0.03 ± 0.02	2.3 ± 0.5	1.4 ± 0.5

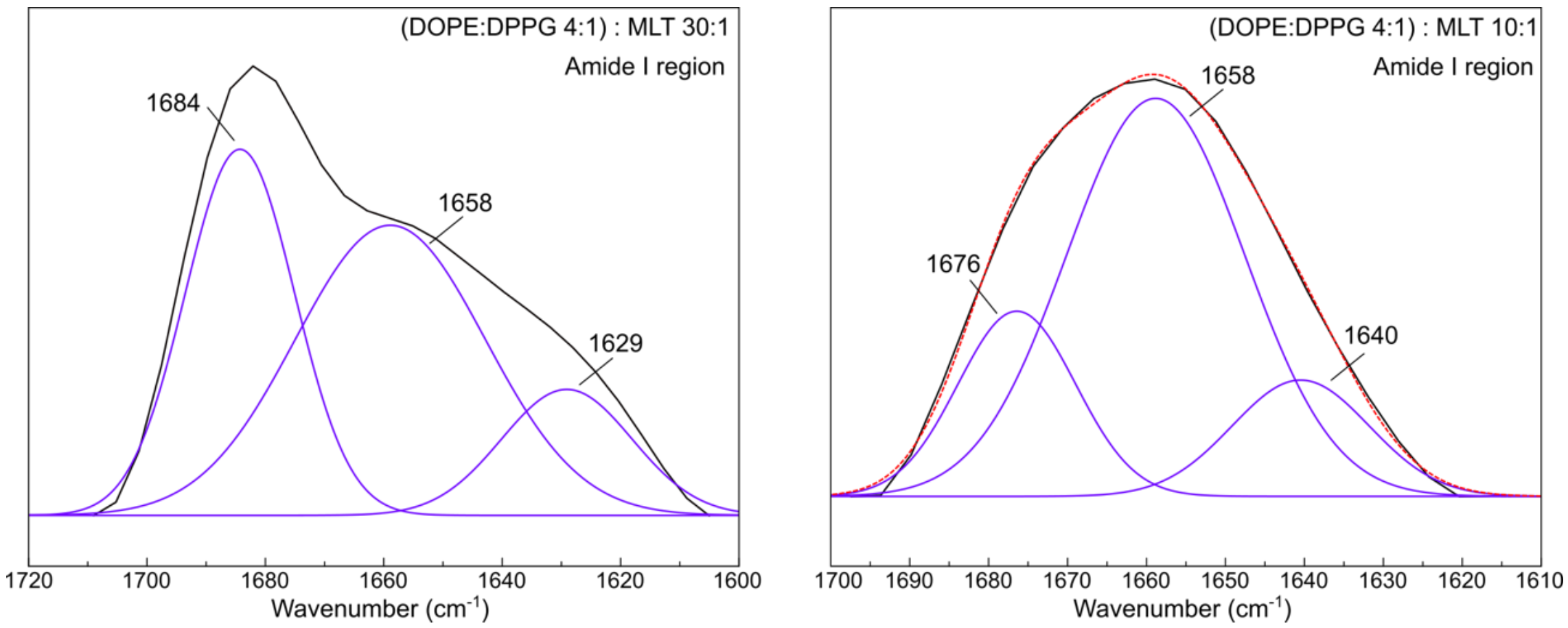

Figure S1. Study of melittin secondary structure through the amide I band in PM-IRRAS. Deconvolution of PM-IRRAS spectra of (DOPE:DPPG 4:1):MLT 30:1 (left panel) and 10:1 (right panel) L:P ratio monolayers on the 5 mM HEPES subphase at a surface pressure of 30 mN m-1, in the 1700-1600 cm-1 range, where the amide I band of amino acid chains is found.

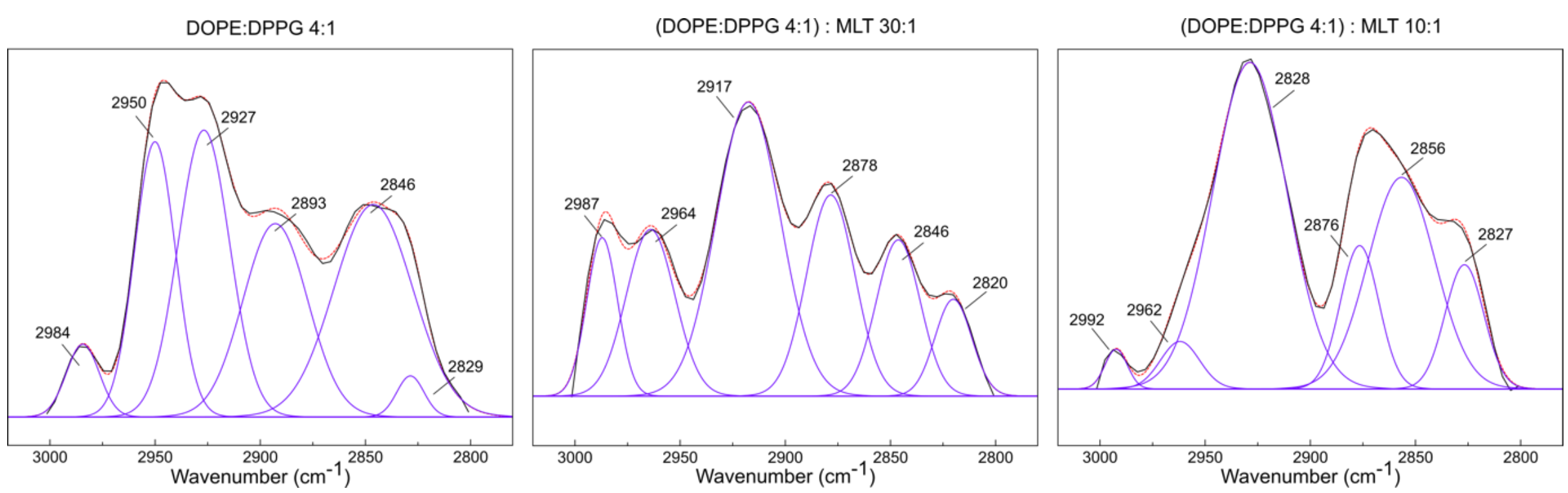

Figure S2. Effect of melittin on acyl chain region of Langmuir monolayers. Deconvolution of PM-IRRAS spectra of DOPE:DPPG 4:1 (left panel), (DOPE:DPPG 4:1):MLT 30:1 (middle panel) and 10:1 (right panel) L:P ratio monolayers on the 5 mM HEPES subphase at a surface pressure of 30 mN m-1, in the 3000–2800 cm-1 range, typical of acyl chain bands.

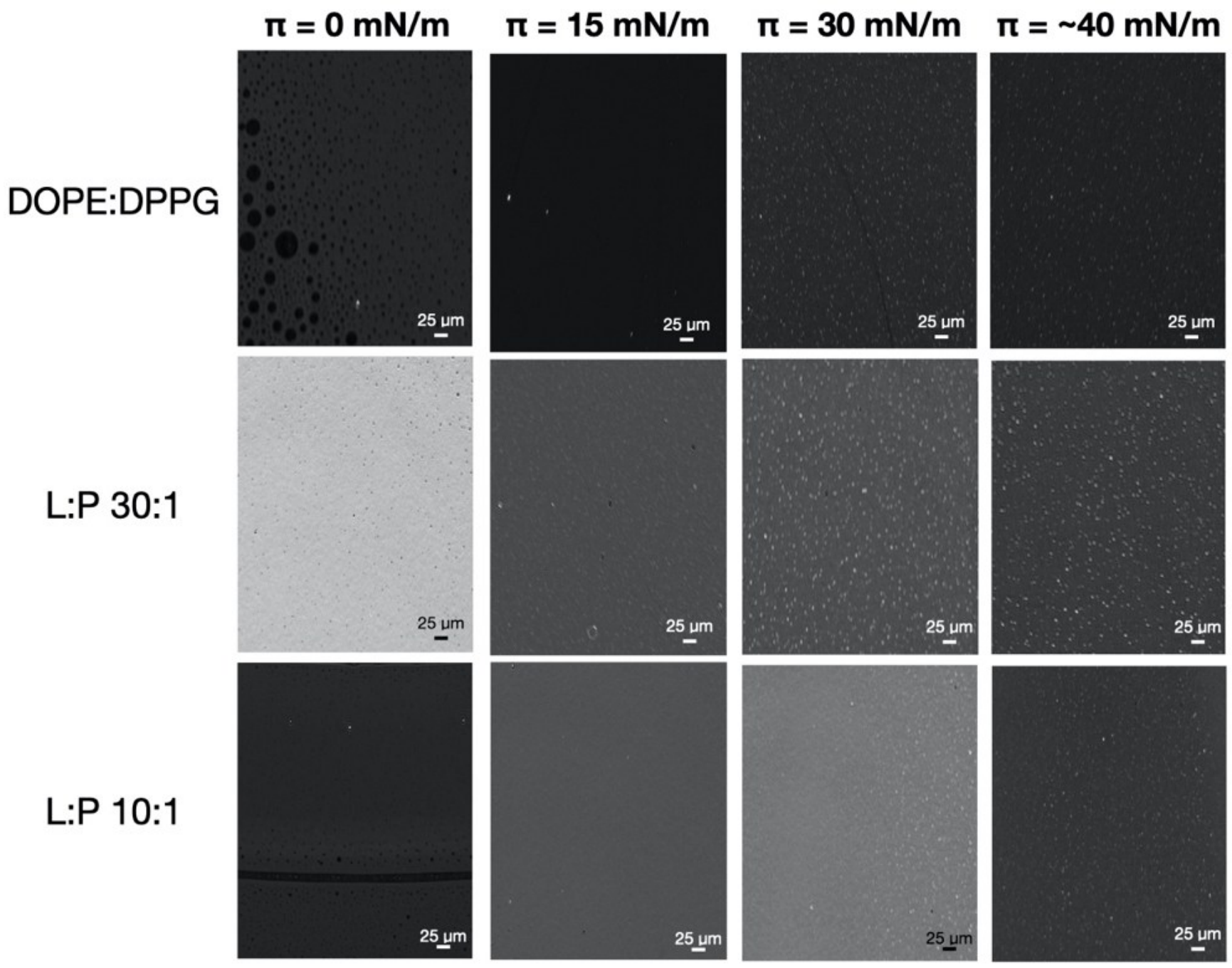

Figure S3. Effect of melittin on Langmuir monolayers. Images obtained by Brewster angle microscopy of Langmuir monolayers of DOPE:DPPG 4:1, (DOPE:DPPG 4:1):MLT 30:1 and 10:1 ratio, at different surface pressure of the compression isotherms (see Figure 1).

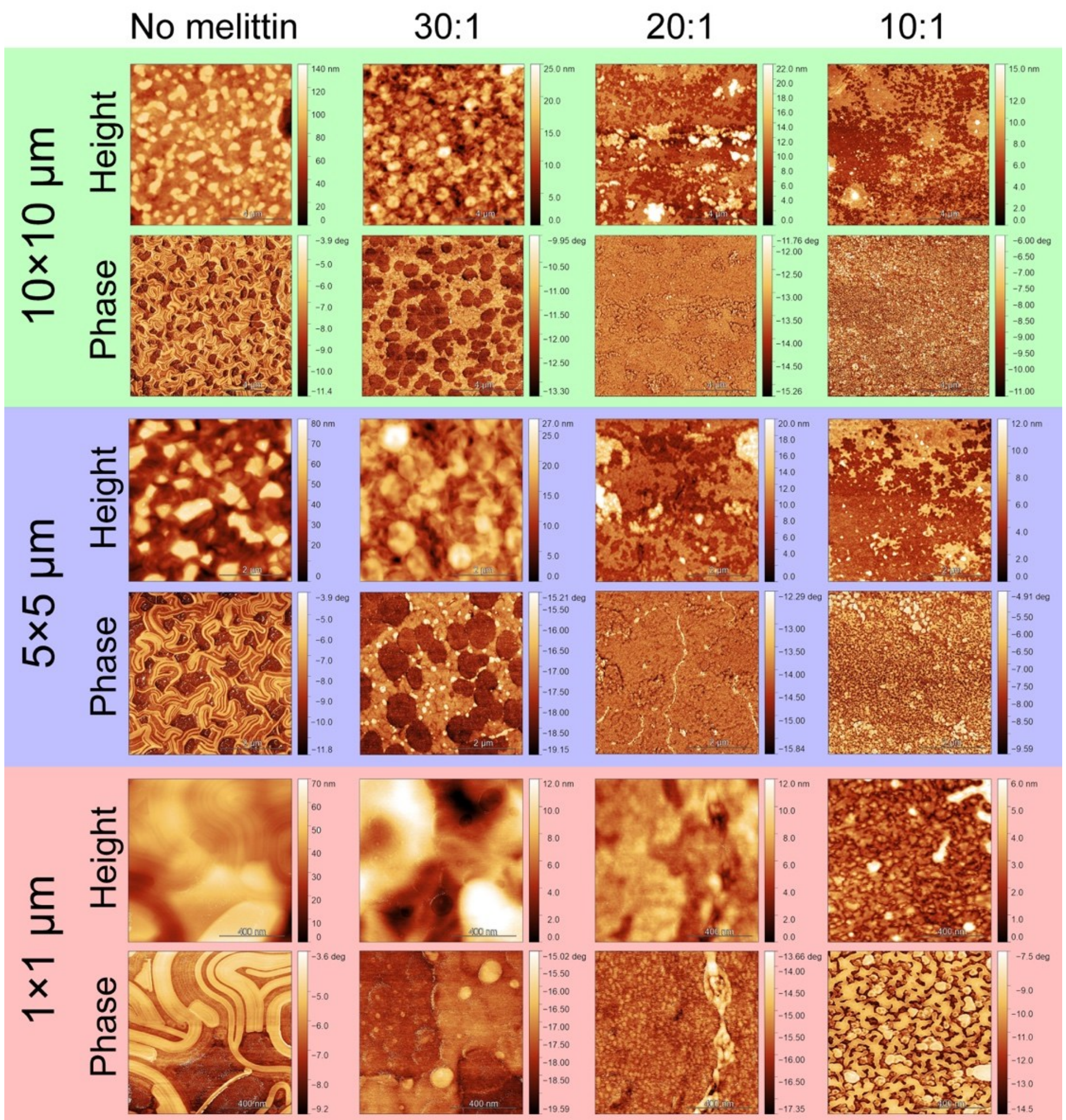

Figure S4. Interaction of melittin with thin lipid films. AFM topographical and phase images of the lipid films of (from left to right) DOPE:DPPG 4:1, (DOPE:DPPG 4:1):MLT 30:1, 20:1 and 10:1 ratio.

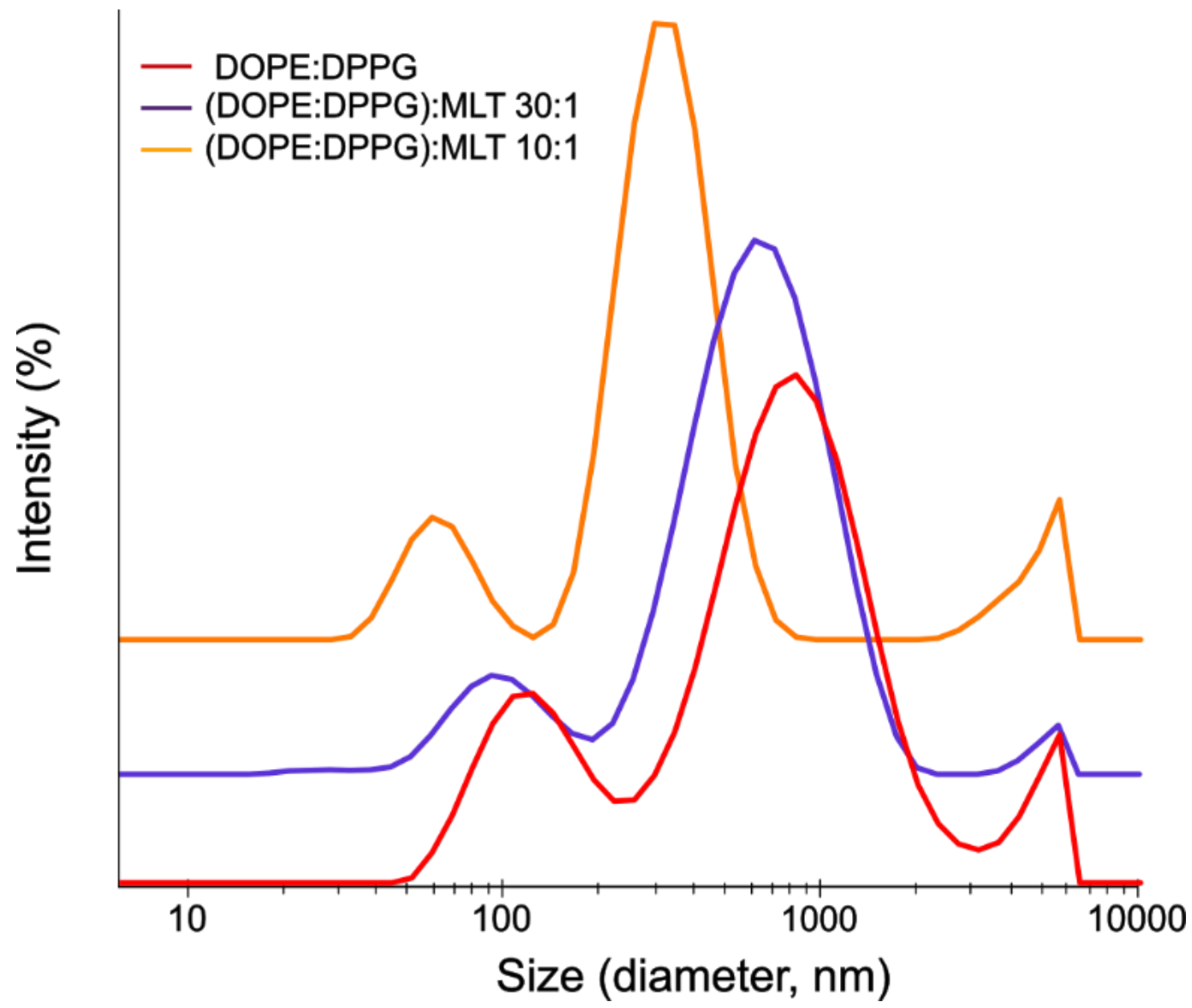

Figure S5. Size distribution changes induced in MLVs by melittin. Size distribution by intensity fraction of DOPE:DPPG 4:1, (DOPE:DPPG 4:1):MLT 30:1 and 10:1 ratio MLVs suspensions.

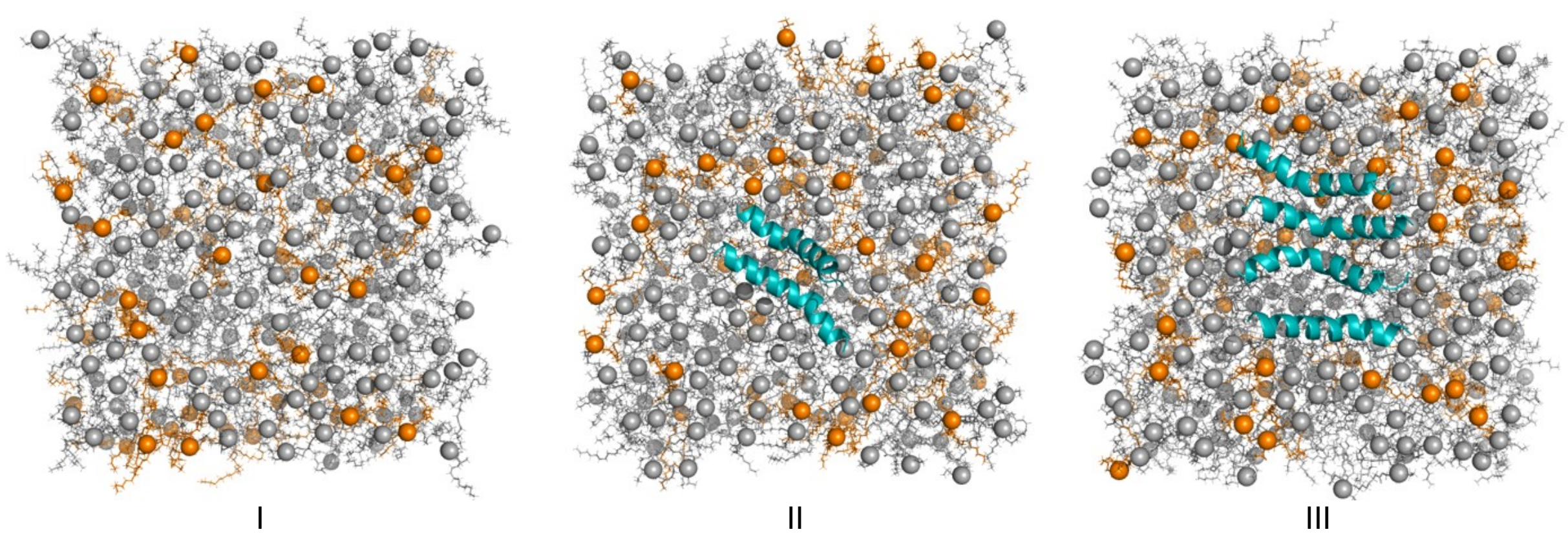

Figure S6. Snapshots from the top view of the initial step (1 ns) of the MD runs for systems I, II, and III. DOPE and DPPG phospholipid are shown in grey and orange, respectively. The P atoms are shown as spheres. Water molecules and ions are omitted for clarity.

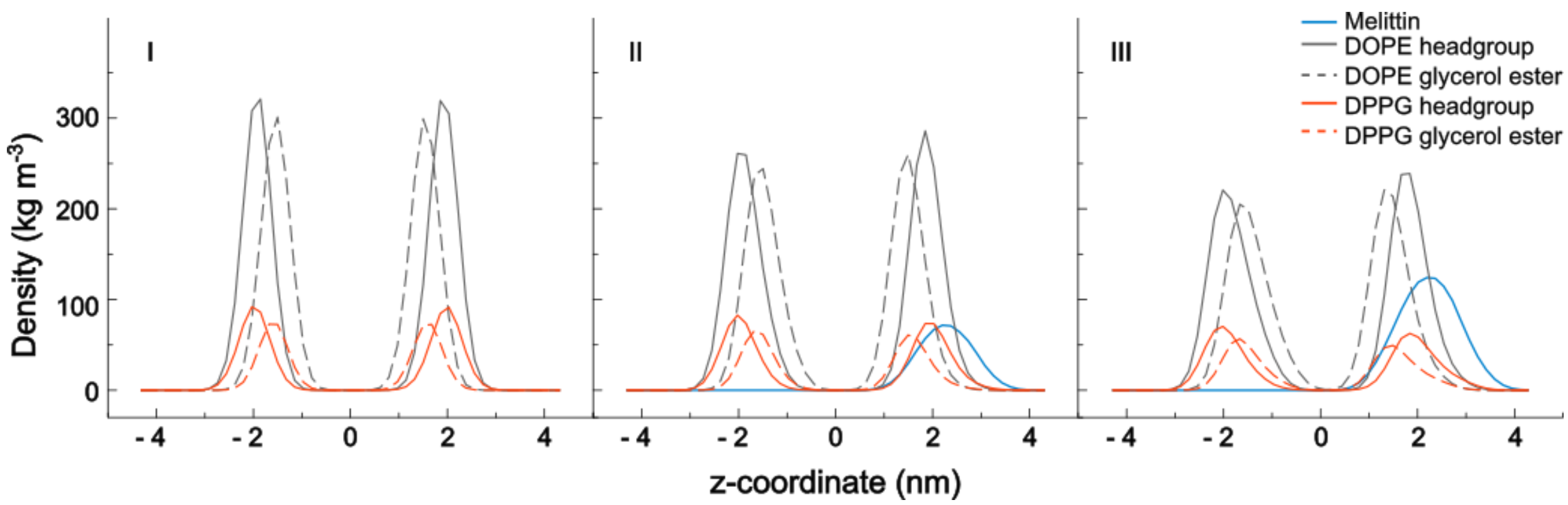

Figure S7. Density profiles for systems I, II and III showing the DOPE (grey) and DPPG (orange) corresponding headgroups (solid line) and glycerol ester groups (dashed line), and the protein (blue). The densities were averaged over 200 ns of sampling.
